# Tick infestations in extensively grazed cattle and efficacy trial of high-cis cypermethrin pour-on preparation for control of ticks in Mvomero district in Tanzania

**DOI:** 10.1186/1746-6148-8-224

**Published:** 2012-11-19

**Authors:** Hezron E Nonga, Adrian Muwonge, Robinson H Mdegela

**Affiliations:** 1 Department of Veterinary Medicine and Public Health, Sokoine University of Agriculture, P.O. Box 3021, Morogoro, Tanzania; 2 Norwegian School of Veterinary Science, P. O. Box 8146, Dep., 0033, Oslo, Norway

**Keywords:** Ecotix^®^, Cattle, Pyrethroids, Tanzania, Ticks, Tick-borne diseases

## Abstract

**Background:**

This study aimed at determining the extent of tick infestations in extensively grazed cattle and assess the efficacy of Ecotix^®^ acaricide (2.5% high cis cypermethrin) in Mvomero district in Tanzania. A total of 1200 Tanzanian short horn Zebu (TSHZ) from two farms in two villages were qualitatively assessed for tick infestations and 40 animals (grouped in 10s from each farm) were separated in their herds and quantitatively examined to establish the tick load. The animals were grouped in treatment regime groups (TxRG 1, 2, 3, and 4), TxRG 1 being the control group was treated with water. Ecotix^®^ was applied on day 0 for TxRG 2, days 0 and 7 for TxRG 3 and days 0, 7 and 14 for TxRG 4 and tick load was monitored for 28 days.

**Results:**

All the animals examined were infested with ticks. The identified ticks were *Rhipicephalus appendiculatus* (55%), *Amblyomma variegatum* (18%), *R. (Boophilus) microplus* (12.9%), *R. (B) decoloratus* (7.1%), *R. evertsi evertsi* (4.4%) and *R. composites* (2.6%). The overall mean (mean ± SEM) tick density on day zero was 63 ± 30 ticks per animal (ranging from 20–160). The mean tick density on day zero was 44.6 ± 25.4, 74.6 ± 30.3, 55.0 ± 26.2 and 77 ± 33.5 for groups one, two, three and four respectively. Post-treatment quantitative assessment of tick burden revealed that the TxRG 1 animals maintained a tick load throughout the study period. A significant decrease in tick load in animals in groups TxRG 2, 3 and 4 (P=0.0001) with increasing frequency of Ecotix^®^ application was recorded. There was however no significant difference in tick reduction between TxRG 3 and 4 (*P=0.0986*). Thus TxRG 3 would be sufficient for the monthly tick treatment and with this regime, the farmer would save up to 2.5 USD per animal during six months of dry season.

**Conclusions:**

The study revealed a high tick infestation among the TSHZ kept in extensive grazing systems in Mvomero district and that when treated with Ecotix^®^ as pour-on preparation using a two application regime per month, the tick control strategy was effective.

## Background

Tick infestation, tick-borne diseases (TBDs) and tsetse-transmitted trypanososmosis constitute are the major problems in livestock production in the Sub-Saharan Africa [1,2]. TBD, namely, East Coast Fever, anaplasmosis, babesiosis and cowdriosis have been reported to affect the optimal livestock productivity in East African region [3,4]. For example, tick-borne diseases constitute over 72% of the annual cattle mortality in Tanzania [5,6]. Apart from acting as vectors for diseases (TBDs), ticks have been recognized as important ectoparasites of livestock. They are bloodsuckers, causing local necrosis which results to low-quality hides [2], secondary bacterial infections [7], lowered productivity in terms of weight gain [8] and milk yield [9]. Studies have shown that ticks of the genera *Rhipicephalus* and *Amblyomma* which are the most important in TBD transmission are distributed in almost all cattle keeping areas in Tanzania [10,11]. Furthermore, the cost of tick control and TBD treatment is increasingly becoming a problem to livestock industry of Tanzania [12]. Notably, in 1997 Tanzania spent up to 54.7 million US$ on tick and TBD control [13], while the total annual national loss inclusive of cattle mortality was estimated to be 364 million US$ [12]. Therefore, because of the immense financial pressure TBD puts on the national livestock productivity, it is imperative that the stakeholders cooperate in designing and recommending effective control strategies for better performance of the industry. Acaricide treatment is the most commonly used method for control of tick-borne pathogens in the tropics [14].

According to Pegram [15] the intensive use of acaricides to control ticks is no more advocated except for “highly productive pure-bred *Bos taurus* dairy cattle”. It is recommended by the “Report of the FAO expert consultation on revision of strategies for the control of ticks and TBDs (Rome, 25–29 September 1989)” that the best method to control or limit the impact of ticks and TBDs is the enzootic stability which can be achieved by different means. However, the approach to ticks and TBDs control by enzootic stability sounds good but may not apply in Tanzania because the prevalence of ticks and TBDs and their associated losses are still very high [16-20]. In 1970s and 1980s the Tanzania government used to subsidise acaricides to farmers but later was stopped because of economic crisis. Many farmers stopped from dipping their animals which lead to an alarmingly increase of the prevalence of tick-borne diseases and their associated losses. Currently, the Ministry of Livestock Development in Tanzania advocates the use of acaricides as the cornerstone means for tick and tick-borne diseases control in the country. Other means of tick control like hand picking, pasture burning, rotational grazing and many others are also encouraged though their practicability still not promising.

The use of acaricides for tick control in Tanzania has a long history. Since the construction of the first dip tank at Mpwapwa in 1905, 2041 dip tanks and 91 spray races had been constructed all around the country [10] but most of them are now dilapidated. The most commonly used acaricides were the organochlorines such as lindane, dieldrin, aldrin, cyclodiene and toxaphene. Due to unacceptable persistence in the environment, tick resistance and chronic toxicity to mammals and birds, the organochlorines were replaced by organophosphates and pyrethroids [21,22]. For example, tick resistance to lindane and dieldrin in 45% out of 189 strains from 10 different tick species in 17 regions of Tanzania has been reported [21]. Unlike organochlorines, the organophosphate compounds are chemically unstable and non-persistent to the environment [23]. The common organophosphates which have been on use in Tanzania include ethion, chlorpyrifos, chlorfenvinphos and coumaphos. However, there have been reports of organophosphate resistance, associated with prolonged use, incorrect strength or wrong concentration of the acaricide [23-25].

On other hand, synthetic pyrethroids like cypermethrin, deltamethrin and cyhalothrin have been reported to be more effective against ticks in the tropics [23,26,27]. These chemicals are favoured over organochlorine because of their efficacy, inherent short half-life and inability to persist in the environment [28]. The introduction of synthetic pyrethroid preparations in Tanzania such as deltamethrin [29], cypermethrin [26] and alpha-cypermethrin [27] which have the ability to control both ticks and tsetse flies is a welcome cost-effective development. Among the synthetic pyrethroid preparations available in the markets are the pour-on preparations, which can be used on individual animals or smaller groups of animals, as well as in areas where water is scarce and where dipping and spraying facilities are not available. Since TBDs is a major problem therefore, it is necessary to document the efficacy and the associated cost benefit of pour-on preparation of synthetic pyrethroids as a tick control alternative in extensive grazing system where most needed. This study was thus aimed to determine the intensity of tick infestations in TSHZ reared in extensive grazing systems. Furthermore, to assess the efficacy of Ecotix^®^ (2.5% high cis cypermethrin) pour-on preparation and cost implications for control of ticks in Mvomero district in Tanzania.

## Methods

### Study location

The study was conducted at Mkula and Melela villages located in the arid areas in southern part of Mvomero district. The two villages were conveniently selected because most of the inhabitants were Maasai pastoralists who use the extensive grazing systems. The villages were easily accessible and had large herds of cattle that had not been dipped for the past ten years. Dipping was not done because of the following; (i) breakdown of the dip tanks, (ii) water scarcity and (iii) high costs of tick control due to cessation by the Tanzanian Government to provide free acaricides to farmers. The important economic losses though not quantified which were incurred by farmers due to tick infestations and TBDs included high morbidity and mortalities, reduced production and reproduction performance in cattle (Hezron E. Nonga, Personal observation, 2009).

Mvomero district is located between 8–10º S and 28–37º E in the Morogoro region, Tanzania. The district varies greatly in topography and climate. Highlands and lowland rainforest are located in the northwest and central-north belt respectively, while the drier woodlands are located in the south. Rainfall distribution in the district is bimodal, with a long wet season from March to May and a short wet season from October to December. The dry seasons in Mvomero district are experienced for six months (June, July, August, September, January and February). The northern area has a humid to sub-humid climate, and annual rainfall ranges from 1500 to 2000 mm while the southern part of the district is much drier, with annual rainfall of up to 600 mm. Average annual temperatures in Mvomero district ranges from 20–30 °C. The study was conducted for four weeks during dry season (July to August) of 2009. During periods of dry seasons, the tick infestations associated with TBDs in Mvomero district are relatively higher as compared to rainy season (Hezron E. Nonga, Personal observation, 2009).

### Study cattle herds

The study animals were the Tanzanian short horn zebu (TSHZ), which were managed under extensive grazing system. Four herds located at a distance of about 10–15 km apart, were conveniently selected from the study villages (two herds in each village). The selection criteria were based on the owners’ willingness to participate in the study. The selected herds had an average of 300 cattle per herd. All the adult animals in the herds were qualitatively examined for the tick infestation. Each animal was examined for presence of ticks on the head, ears, neck, belly, back, legs, perineum and tail. Preliminary identification of ticks (based on body morphology and colour) was done while the ticks were still attached on the animal body. Presence of one or more ticks on an animal was recorded as a positive case.

### Quantitative analysis of tick infestation

After the animals were examined for presence of ticks, 10 adult cattle from each herd were selected for quantitative tick assessment and subsequently were used in the Ecotix^®^ treatment trials. The animals were selected based on the following criteria: (i) had many visible ticks on the skin (positive cases of tick infestation), (ii) the age was one year and above, (iii) had a weight range of 100 to 200 kg and, (iv) there was no history of application of acaricide. Animals with the stated criteria were ear tagged and separated from the rest in each study herd except the control group (TxRG 1). A total of 40 animals were selected and allocated in treatment regimes groups (TxRG 1–4) according to their herds of origin. The 10 animals in each of the treated groups (TxRG 2, 3 and 4) were separated from the rest and were being grazed separately during the day time but kraaled with rest of the animals in the herd during the night. The experimental animals in all the four groups were being grazed in terrain of pasture of similar characteristics since the interval from one study village (Melela) to the other (Mkula) was almost 15 – 20 km which did not give any significant differences in climate and general weather conditions. Animals in TxRG 1 and 4 were from the herds in Mkula village while those in group 2 and 3 were from Melela village. All the selected animals were female (cows) since cows constituted more than 95% of the animals in the study herds.

For quantitative tick assessment, individual animals in each group were restrained, cast down and examined for ticks. Each animal was examined by thorough checking and where required combing, all the body regions beginning from the head and ears, followed by the neck, belly, udder, back, limbs, perineum and tail. Quantitative examination of each animal involved tick counting on the whole body [30,31]. With the aid of portable magnifying lens, adult and immature ticks on the animals were preliminary physically identified (based on body morphology and colour) as described by Soulsby [32] and Walker et al. [33]. After the preliminary field identification of ticks [32,33], further identification in the laboratory using a sterio microscope was done. A maximum of 2–3 ticks for each identified category were collected and stored separately in tubes of ethanol 70%. The samples were sent to the laboratory at Faculty of Veterinary Medicine, Sokoine University of Agriculture for microscopic confirmation. Morphological and structural characterization of ticks was carried out using a stereoscopic microscope with the aid of identification guides of Soulsby [32] and Walker et al. [33]. The identified ticks were not separated based on sexes and location of attachment on the body of an animal.

### Ecotix® treatment and monitoring of the animals

The selected 40 animals (TxRG 1–4) were involved in the Ecotix^®^ pour-on preparation (Farm Base, Tanzania Ltd) treatment trials. Ecotix^®^ contains 2.5% of high-cis cypermethrin active ingredient. The treatment trial was conducted as described by Sajid et al. [31] with some modifications. In TxRG 2, 3 and 4 animals were treated with Ecotix^®^ according to manufacturer instructions while animals in TxRG 1 were treated with water (served as a negative control). Briefly, Ecotix^®^ was applied along the spinal from the head to the tail at the recommended dose (1 mL per 10 kg body weight). Day 0 was the day that the cattle were first examined for tick infestation and Ecotix^®^ was applied to the animals in groups TxRG 2, 3 and 4. The acaricide was applied on day 0 for TxRG 2, days 0 and 7 for group 3 and days 0, 7 and 14 for TxRG 4. Tick load in the control group was monitored along-side with the treatment TxRG. Tick load on animals was quantitatively monitored through physical counting on the whole body [30,31]. Tick infestation was monitored in the control and treatment groups for a period of 28 days as from day 0, 1, 4, 7, 10, 14, 17, 21 and 28. Number of ticks and effectiveness of treatment duration, was calculated from the data for each group.

### Data analysis

The prevalence of ticks was determined using the following formula [34]:

$$\text{Prevalence}\;\left(\%\right)=\frac{\text{Number of existing cases during specified time period}\;\text{x}\;100}{Population\;at\;risk\;during\;that\;specified\;time\;period}$$

Population at risk during that specified time period

The data was entered in Microsoft Excel^®^ spreadsheet. After validation the data were then transferred to Stata (stata/SE 10 for Windows, StataCorp, College Station, TX) for statistical analysis. A mean tick count in box graph plot was used for graphical analysis. Because of differences in the number of ticks that were observed on animals on day 0 in different groups, before plotting, all means for tick counts were normalized by computing percentage of number of ticks counted relative to tick counts on day 0 in each group. A robust Poisson regression model was used to show the effect of the different treatment regimes on the total tick count.

### Ethical considerations

The permission to carry out this study was granted by the Morogoro municipal livestock officer and ethical approval for the study was given by the Ethical Committees of Sokoine University of Agriculture (Morogoro, Tanzania). Verbal consent was obtained from each of the participating heads of households after explaining the purpose and importance of the study prior to commencement of the experiments.

## Results

### Tick infestation in cattle

All the examined animals (n=1200) from the 4 study herds were infested with different species of ticks. However, not all the tick species identified, were present in all the animals. The tick species identified on the animals are shown in Table 1. The most predominant identified tick species was the brown ear tick *Rhipicephalus appendiculatus*. Other identified tick species included *A. variegatum, R. (B) microplus* (formerly called *Boophilus microplus*)*, R. (B) decoloratus* (formerly called *Boophilus decoloratus*)*, R. evertsi evertsi* and *R. compositus.* The overall mean (mean ± SE) tick density was 63 ± 30 ticks per animal with a count range between 20 and 160. The mean tick count on day 0 between the animal TxRG was as follows: 45 ± 25, 75 ± 30, 55 ± 26 and 77 ± 34 for groups one, two, three and four respectively. 

**Table 1:** Total number of tick species identified in 40 cattle on day 0 of the experiment

**Tick species**	**Total number of ticks counted**	**Percent**
*R. appendiculatus*	1354	55.0
*A. variegatum*	394	18.0
*R. (B) microplus*	318	12.9
*R. (B) decoloratus*	175	7.1
*R. evertsi evertsi*	109	4.4
*R. compositus*	63	2.6
Total	2462	100

### Results on ecotix® treatment trial

**Figure 1:**
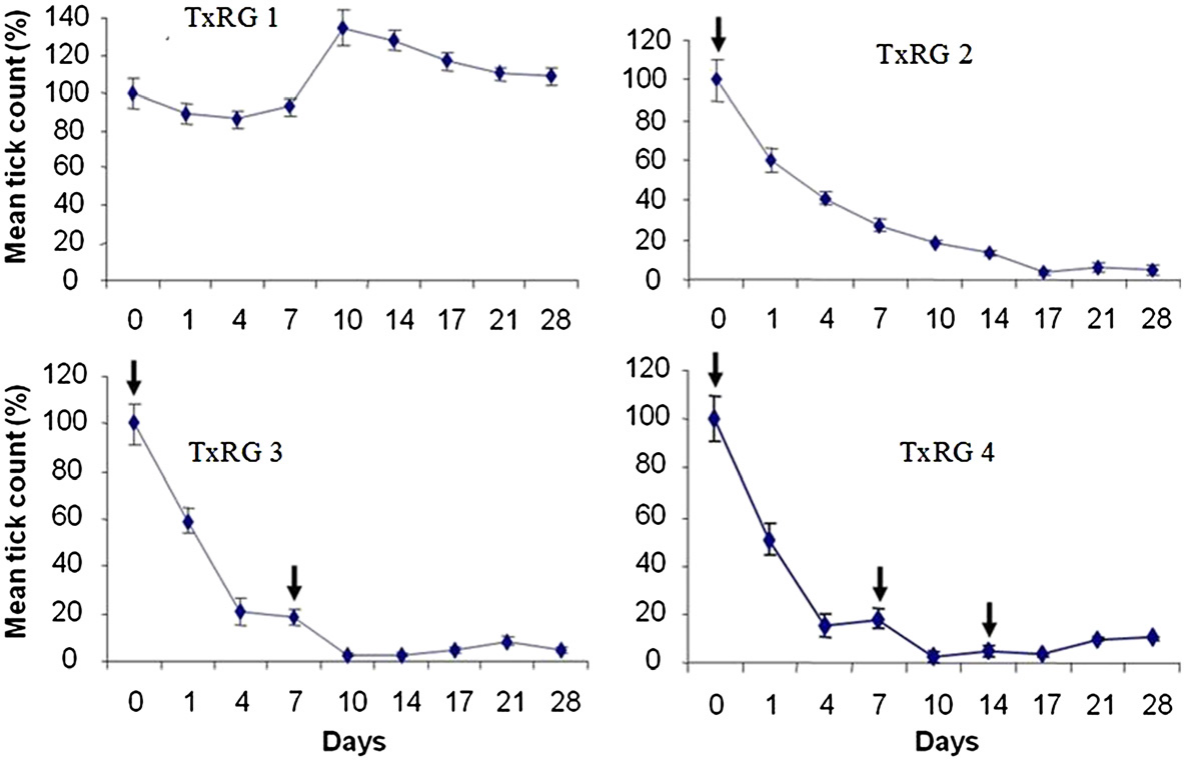
**The mean ± SEM for tick counts in different treatment regime groups (TxRG) and TxRG 1 is the control group**. Ecotix^®^ was applied on day 0 for TxRG 2, days 0 and 7 for TxRG 3 and days 0, 7 and 14 for TxRG 4. Tick counting was done on day 0, 1, 4, 7, 10, 14, 17, 21 and 28. The results are presented as percentage of change in tick counts from day 0 to 28. Note that the tick counts in the control group (TxRG 1) on average remained constant. As a post Ecotix^®^ application effects on day 0 in TxRG 2, tick count decreased from 100% to 5%, in TxRG 3, the tick counts decreased from 100% to 4% and in TxRG 4, the tick counts decreased from 100% to 10%. The second dosage on day 7 in the TxRG 3 and TxRG 4 dramatically reduced the total tick count in these two groups compared to the TxRG 2. Spontaneously, the tick loads in TxRG 3 and TxRG 4 started to increase as noted on day 14, 17, 21 and 28 of counting. No difference in the total number of tick between the TxRG 3 and TxRG 4. Represents treatment days.

The post-treatment quantitative assessment of tick burden revealed that untreated animals (control group) maintained a tick infestation throughout the study period with a slight increase recorded on day 10 and 14 but later normalized (Figure 1). The number of ticks in the control group (TxRG 1) average remained constant. Following application of Ecotix^®^ pour-on preparation on day 0 in TxRG 2, tick count decreased from 100% to 5%, in TxRG 3, the tick counts decreased from 100% to 4% while in TxRG 4, the tick counts decreased from 100% to 10% by day 28 (Figure 1). 

Since all the three test regimes; single treatment (TxRG 2), double treatment (TxRG 3) and triple treatment (TxRG 4) received the same initial concentration of Ecotix^®^ pour-on preparation, the fall in tick count had the same characteristic but the count at day 7 was different since these groups had a different total tick count at day 0. The second dosage on day 7 which was applied to the double and triple treatment regime groups had a dramatic reduction in the total tick count between these two groups (TxRG 3 & TxRG 4) compared to the single treatment regime group (TxRG2) (Figure 1). The mean tick count was 2% and 3% for groups TxRG 3 and TxRG 4 respectively. Spontaneously, the tick loads in TxRG 3 and TxRG 4 started to increase as noted on day 14, 17, 21 and 28 of counting. This spontaneous increase seemed not to be affected by the third Ecotix^®^ which was applied on day 14 TxRG 4 group. Furthermore, given that all the four groups had different total tick number at day zero, a robust Poisson regression was used to eliminate that effect. It was found that there was no significant difference on total tick number when double (TxRG 3) and triple (TxRG 4) Ecotix^®^ treatments were compared (double vs triple treatment P=0.0986). However, a significant difference (P=0.0002) was observed when single vs double treatment and single when compared to triple treatment (P=0.0001).

### Economic analysis of ecotix®

During the high tick challenges, the manufacturer recommends a regime of three times applications of Ecotix^®^ pour-on to cattle per month. Based on the quantitative treatment trials, a simple economic evaluation which applies for these geographical settings was computed extrapolating the analysis to all six months of dry season which has a relatively high tick challenge is described below: Doses of 1 mL of Ecotix^®^ pour-on applications per 10 kg body weight are required. The average weight of the TSHZ that met our selection criterion for study inclusion weighed 150 kg, so 45 mL would be needed in a month making a total of 270 mL per animal during the 6 months of dry season. The current selling price of 250 mL bottle of Ecotix^®^ in Tanzanian shillings (Tshs) is 10, 000= (equivalent to 6.8 USD) so each mL costs 40 shillings and the cost per animal during the six months of heavy tick infestation would be 10,800= (7.5 USD). From our findings, if TxRG 3 is used, only 180 mL of Ecotix^®^ would be used per animal per six months costing 7, 200 shillings. In economic terms, the farmer saves Tshs 3, 600/= (2.5 USD) per animal per six months by adopting double Ecotix^®^ application per month (exchange rate 1 USD = 1, 480 Tshs). Note that this economic analysis applies only in areas like southern parts of Tanzania including Mvomero district which have similar climatic conditions and tick challenges especially during the dry season.

## Discussion

This study demonstrates that different species of single and multi-host tick species were prevalent in the TSHZ cattle which were managed under extensive system. The study shows five different species of ticks, which were predominated by *R. appendiculatus*, a well-known vector for *Theileria parva* which causes East Cost Fever in cattle. *R. appendiculatus* inhabits a wide range of East African environments [35] and has been recorded from different parts of the Tanzania mainland [10,21]. The favourable climatic conditions for survival and multiplication are cool moist places, but it can tolerate relatively arid conditions as in case of many areas inhabited by pastoralists in Tanzania. Branagan [36] reported that presence of favourable temperature, humidity, rainfall and appropriate hosts as important factors for the endemicity of *R. appendiculatus* in a locality. Almost all the above listed factors are favourable in Morogoro and this may account for *R. appendiculatus* predominance. Predominance of *R. appendiculatus* may also be due to the fact that cattle are widely believed to be the primary host for this tick [37,38]. The other species, *A. variegatum* (vector for *Cowdria ruminantium*), *R.(B) microplus* (vector for *Babesia* spp and *Anaplasma* spp) *R. (B) decoloratus* (vector for *Babesia* spp.) and *R. evertsi evertsi* (vector for *Anaplasma* spp.) occurred at a relatively low numbers suggesting that they are less abundant under field conditions. Our findings on the general spectrum of ticks were similar to those of other authors in Tanzania [10,11,25,38]. Considering previous studies on tick infestation in domestic ruminants and results of current study, unless adequate and sustainable tick control measures are put in place, tick infestation will remain the main drawback to livestock industry in Tanzania.

Furthermore, studies reports that there is a co-existence between the geographic distribution of *R. (B) decoloratus, A. variegatum* and *R. appendiculatus*, in the absence of tsetse or any other barrier to cattle distribution, *R. (B) decoloratus* and *A. variegatum* tend to be centered on the same foci as *R. appendiculatus*[10,11,25]. The reason behind can be both the climate and host factors for their survival and multiplication. Generally, the tick challenge recorded during this study could be attributed to the more conducive conditions for tick proliferation, survival and lack of tick control programs in most pastoralist communities in Tanzania [39].

Lack of tick control measures on animals grazed in infested areas and the poor animal husbandry practices may be other factors for the observed high tick loads. Moreover, the limited scope of awareness regarding the impact of ticks, non existence of acaricide uses and lack of adequate veterinary infrastructure for access by pastoral society in the area may also account for the widespread existence of tick species in the area. This high prevalence of ticks predisposes the animals to TBDs which would ultimately lead to losses due to reduced productivity and increased animal mortality. Indeed, a study by Swai et al. [38] in Maasai pastoralists in Tanzania reported that TBDs were the main cause of low productivity and cattle mortality. However, some authors [15,40,41] believe that animals which are not treated with acaricide in tick infested endemic areas tend to achieve an endemic stability to TBD, where adult cattle acquire immunity against subsequent haemoparasitic infections and severe diseases only occur in calves and young animals. In reality, this may not be practical and needs more research. For cowdriosis, babesiosis and anaplasmosis, a high tick challenge and high prevalence of TBDs are actually the conditions allowing the setting up of the enzootic stability. However, some researchers are not sure that such enzootic stability can prevail for ECF, because of the very high pathogenicity of the disease in cattle including the local breeds. Since *R. appendiculatus*, the vector of ECF was the main tick in the examined herds, this could prevent the setting up of enzootic stability. Indeed, a study by Silayo et al. [16] reported that in absence of acaricide use in cattle kept under extensive grazing system in Tanzania, calf mortalities due to TBDs in particular ECF can reach up to 80%. Calves are infected naturally with haemoparasites within four weeks of birth and in the absence of acaricide application, their health status decline severely and succumb to high mortality rates [17,42]. In addition, in absence of routine use of acaricides, increased morbidities, reduced production and reproduction performance associated with high mortalities in adult cattle has also been reported in different places [18-20,43].

Therefore, the results of present study warrant for immediate tick control measures. The frequent use of efficacious acaricides as a control measure may avert major cattle losses through deaths and loss of productivity which were reported by the farmers during the study period. Also, there should be an effort to educate the famers about the significance of the tick infestations and the best approach to control measures.

According to the Tanzania Ministry of Agriculture [44], an acaricide is considered to be adequate when its efficacy is 95% or higher. Ecotix^®^ as a pour-on preparation showed that it has high acaricidal effects as it had cleared off almost 95% of the wide range of hard multi-host ticks in cattle in the period of 10 days post-treatment. The second treatment (Figure 1) which was applied in TxRG 3 and TxRG 4 gave more better results as it further reduced the tick loads from 20% and 19% to almost 2% and 3% respectively. This lead to our conclusion that repeated treatment of Ecotix^®^ to cattle at two weeks interval gives a more better results in controlling ticks as it showed a significantly low tick loads compared to single treatment. However, the spontaneous decrease in tick count observed in TxRG 2 which had received single Ecotix^®^ could not warrant recommending a single treatment and this needs further investigations. The observed slight decrease of tick load in TxRG 4 post third Ecotix^®^ treatment and thereafter a spontaneous increase of tick count as from day 17 to 10% by day 28 could be not be accounted for but may be caused by extent of tick challenge and management factors. Generally, these results are also in agreement with work done by Msolla [26] who found that Ecofleece, a 10% w/v cypermethrin preparation was 99% effective in the control of both single and multi-host ticks up to two weeks post treatment. Similarly, Mbise et al. (unpublished report) reported that alpha–cypermethrin controlled significantly both the immature and mature ticks. Elsewhere, Bicalho et al. [45] reported that the high cis-cypermethrin had a 75% inhibition of *R. sanguineus* eggs from hatching while it had 100% larval, nymphal and adult tick mortality. We therefore recommend the use of Ecotix^®^ pour-on preparation in the routine control of ticks and TBDs in Tanzania.

Furthermore, the study found that Ecotix^®^ a pour-on preparation was able to control ticks by double treatment per month different from the three treatment recommended by the manufacturer. For economic reasons, double treatment is cost effective for control of ticks. The high-cis cypermethrin are reported to have a persistent effect and are generally considered to be an improvement over other insecticides relative to their environmental impact when applied at insecticidal doses [46].

Economically, with double treatment regime of Ecotix^®^ a pour-on preparation, the farmer can save up to 2.5 USD per animal per each six months of dry season when the extent of tick infestation is relatively high. Based on the common average herd size of up to 5,000 owned by a pastoral household in Tanzania, the farmer may save up to 12,500 USD in six months of dry season.

## Conclusions

From the findings of this study, it is concluded that cattle in Mkula and Melela villages in Mvomero districts were infested with different species of ticks at varying intensities. A 2.5% high-cis cypermethrin pour-on showed high efficacy against tick infestations in TSHZ cattle managed under extensive system in this area. Furthermore, 2.5% high-cis cypermethrin preparation can be applied to cattle grazed under extensive management system twice per month at two weeks interval rather than thrice per month as recommended by the manufacturer. With this Ecotix^®^ treatment regime, the farmer is able to save 2.5 USD per animal during the six months of dry season.

## Competing interests

This study was funded by Farm base Ltd of Dar es Salaam, Tanzania which is the manufacturer of Ecotix^®^ pour-on preparation.

## Authors’ contributions

HEN: Developed the concept, wrote the protocol, collected the data during field work, drafted the manuscript and final proof reading of the manuscript before was submitted to the journal. AM: Statistical analysis of all the data, interpretation and description of analysed data, read and corrected the manuscript on technical and language part. Edited the manuscript before was submitted to the journal.RHM: Planned and set the experiment, facilitated in design and coordination of the experiments during field work, advised on the manuscript formatting, technical advice on different kinds of acaricides and their use in Tanzania. Edited the manuscript before was submitted to the journal. All authors read and approved the final manuscript.
